# Photodynamic therapy monitoring with optical coherence angiography

**DOI:** 10.1038/srep41506

**Published:** 2017-02-02

**Authors:** M. A. Sirotkina, L. A. Matveev, M. V. Shirmanova, V. Y. Zaitsev, N. L. Buyanova, V. V. Elagin, G. V. Gelikonov, S. S. Kuznetsov, E. B. Kiseleva, A. A. Moiseev, S. V. Gamayunov, E. V. Zagaynova, F. I. Feldchtein, A. Vitkin, N. D. Gladkova

**Affiliations:** 1 Nizhny Novgorod State Medical Academy, Minina Square 10/1, 603005 Nizhny Novgorod, Russia; 2 Institute of Applied Physics Russian Academy of Sciences, Ulyanova Street 46, 603950 Nizhny Novgorod, Russia; 3 Republican Clinical Oncology Dispensary, Gladkova F. Street 23, 428000 Cheboksary, Russia; 4 University of Toronto and University Health Network, 610 University Ave., Toronto, Ontario, M5G 2M9, Canada

## Abstract

Photodynamic therapy (PDT) is a promising modern approach for cancer therapy with low normal tissue toxicity. This study was focused on a vascular-targeting Chlorine E6 mediated PDT. A new angiographic imaging approach known as M-mode-like optical coherence angiography (MML-OCA) was able to sensitively detect PDT-induced microvascular alterations in the mouse ear tumour model CT26. Histological analysis showed that the main mechanisms of vascular PDT was thrombosis of blood vessels and hemorrhage, which agrees with angiographic imaging by MML-OCA. Relationship between MML-OCA-detected early microvascular damage post PDT (within 24 hours) and tumour regression/regrowth was confirmed by histology. The advantages of MML-OCA such as direct image acquisition, fast processing, robust and affordable system opto-electronics, and label-free high contrast 3D visualization of the microvasculature suggest attractive possibilities of this method in practical clinical monitoring of cancer therapies with microvascular involvement.

Mechanisms of tumour tissue damage in photodynamic therapy (PDT) are still a subject of scientific research^1^,^2^. Three fundamental mechanisms of PDT effects are tumour cells damage, blood vessels damage and immune response^3^,^4^,^5^. The combination of these mechanisms is thought to give pronounced long-term effect. The contribution of each mechanism to total response depends on characteristics of the photosensitizers (PS), structure of the tumour, tumour vascularization, tissue oxygen content, interstitial and intracellular distribution of the PS, the type and duration of inflammation and immune response, and the parameters of treatment delivery such as total light exposure, PS delivery to treatment commencement interval, and fluence rate. Moreover, many of these are complexly interlinked and depend on each other^4^.

The temporal dynamics are also complex in that each type of reaction has its own characteristic time. The blood vessels response is typically the earliest, developing within minutes/tens of minutes from the beginning of the treatment, and can in fact be detected even during the PDT process itself^3^,^6^,^7^. Tumour cell damage/necrosis typically develops next, 1-2 days after PDT. The immune reaction is the slowest process, which can take from a few days to weeks to evolve to the observable stage^6^.

To improve PDT effect on tumours, minimize the tumour regrowth, decrease collateral normal tissue damage and improve cosmetic outcomes, it is important to develop approaches to a real-time noninvasive monitoring of tumour response to optimize treatment delivery parameters. There are ‘explicit’ and ‘implicit’ classes of PDT dosimetry. The complicated interaction between light, photosensitizer and tumour microenvironment in PDT makes ‘explicit’ dosimetry a dynamic and difficult problem^2^,^8^,^9^. Alternatively, several researches have used photobleaching of photosensitizer for ‘implicit’ PDT dosimetry^10^,^11^. Singlet oxygen luminescence monitoring has also been investigated^12^. The advantages and limitations of each approach have been examined^2^,^8^. Given the early time course and dynamic nature of tumour and normal tissue microvascular treatment response, angiographic imaging during/following PDT offers an attractive possibility.

The vascular-targeted regime is mainly responsible for some of the more successful clinical implementations of PDT today^13^,^14^. Stasis of blood and extravasation, vascular occlusion and microcirculatory changes are considered dominant microvascular biological response to PDT^15^. The degree of vascular response is in part dependent on concentration of PS circulating in the blood or accumulated in vessels’ endothelium at the time of laser exposure^4^.

Blood flow dynamics can be studied with laser speckle interferometry^16^, fluorescent contrast agents^17^, photoacoustic imaging^18^, and optical coherence tomography (OCT)^7^,^19^,^20^,^21^,^22^. It has been demonstrated that photoacoustic imaging could be potentially used to guide PDT and other phototherapies using vascular changes during treatment to optimize treatment protocols, by choosing appropriate types and doses of photosensitizers, and doses of light^23^. Changes in blood oxygen saturation measured by photoacoustic imaging suggested criteria of PDT success^24^. OCT-derived microvascular response metrics have also been reported^25^. However, it is clear that much additional work is needed to determine sensitive and robust microvascular predictors of PDT response.

In this study, we hypothesized that the early tumour response to PDT may manifest itself as the disappearance of microvascular network on MML-OCA images due to vascular damage and blood flow disruption. The study was performed on the ear model of the murine colon carcinoma CT26. Tumour microvascular and growth were assessed during the 7 days following Photoditazin (a clinically-approved chlorine E6 photosensitizer) PDT, and histopathology with hematoxylin-eosin (H&E) staining was performed at the final time point to analyze cellular and vascular damages. Accumulation of the photosensitizer in the tumour and its photobleaching dynamics were also detected by fluorescence imaging *in vivo*. The key goal of the study was to find criteria to predict early tumour response to PDT based on MML-OCA visualization of microvascular network within 24 hours after treatment.

## Results

### Tumour volume changes after PDT

**Figure 1:**
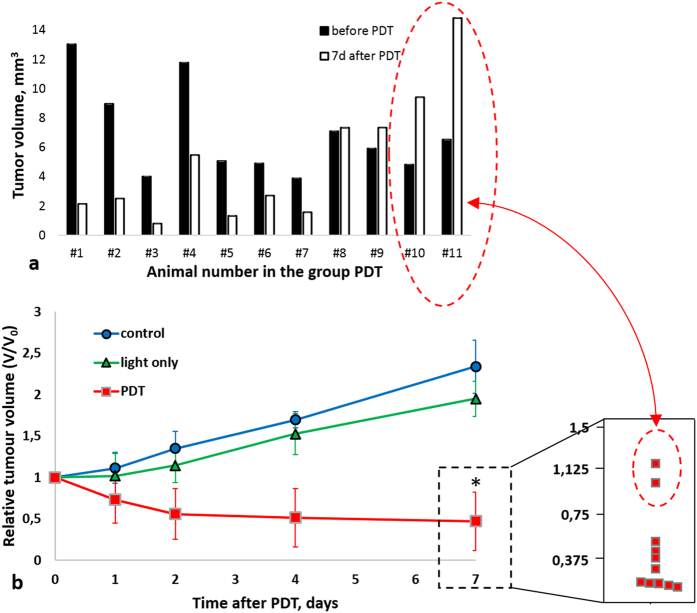
Effect of PDT on CT26 tumour growth. (**a**) Individual tumour volumes in the PDT group. Seven of eleven tumours regressed after PDT; in two animals (#8, 9, there was tumour growth arrest but no volume decrease, and in two other cases (#10, 11) tumour growth continued despite PDT (**b**) Monitoring of relative tumour volume changes for treated (n = 11), light only (n = 5) and untreated (n = 5) control groups. Data are shown as mean ± SD. Tumour growth was overall inhibited by PDT in the treatment group, showing pronounced volume decrease. *Statistically significant difference from light-only and untreated groups, *p* ≤ 0.05. Inset demonstrates distribution of eleven tumour volumes at 7 days post PDT. Despite strong (and statistically significant) overall decrease in tumour volumes in the PDT group, the distribution of individual tumour volumes revealed the presence of two moderate responders and two non-responders in this cohort; see text for a discussion on this finding.

Figure 1a demonstrates that tumour volumes just before PDT varied broadly, ranging from 4 to 13 mm^3^, both between and within the animal groups. Still, 9 of 11 tumours (#1–9) showed significant volume decrease (#1–7) by 7 days after PDT, with their TGI metric (it is defined in Materials and Methods in section Tumour growth study) varying from 147% to 165% (Fig. 1, Table 1). Two of 11 tumours remained ~ same size (#8,9, TGI 142% and 135%), and 2 continued to grow (#10, 11, TGI 97% and 81%). Nevertheless, on day 7 the PDT-treated cohort had statistically significant smaller volumes compared to untreated controls and to tumours exposed to light only. Conversely, in the untreated and light only groups, tumour volumes approximately doubled (n = 5 in each cohort, Fig. 1b). Exposure of tumours to light only resulted in insignificant inhibition of the tumour growth; TGI in light-only group was 26 ± 2%. This slight decrease in tumour growth rate, although not statistically significant, may be caused by the generation of reactive oxygen species (ROS) as a result of illumination of endogenous fluorophores^26^, or by minor thermal effects^27^.

### Histological examination

**Figure 2:**
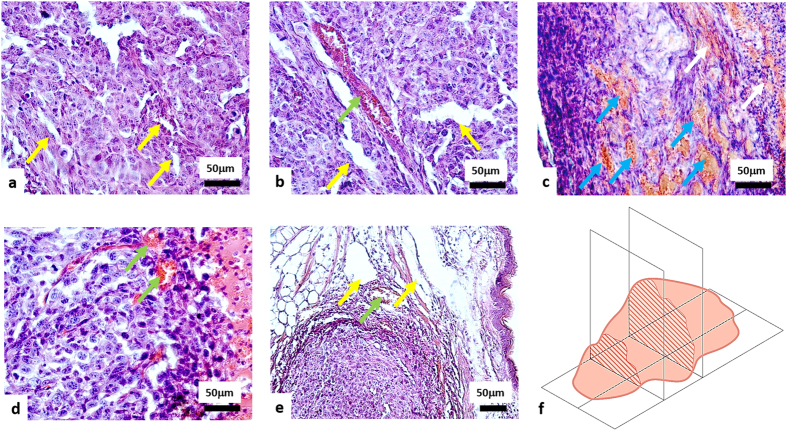
H&E histology of CT26 tumours 7 days after PDT. (**a**) Untreated tumour; (**b**) light only group; (**c**) tumour after PDT with severe microvascular damages; (**d**) tumour after PDT with weak microvascular damages; (**e**) tumours after PDT with severe microvascular damages in the tumour and weak microvascular damages on the border with normal tissue. Yellow arrows indicate undamaged vessels; green arrows - hyperemia; blue arrows – thrombosis; white arrows - hemorrhage. (**f**) Schematic of the orientation of the histological sections. Trends of observed tissue changes were consistent throughout the tumour extent (i.e., similar in its central parts and at margins).

Tumours of untreated and light only groups exhibited 80–100% viable (not damaged) tumour cells. The tumour tissue had a dense structure and consisted of polymorphic cells with diameter varying between 10 and 20 μm and round or oval nuclei. The slightly basophilic cytoplasm formed a thin ring around the nucleus. Microvascular damages were not seen (Fig. 2a). Histological examination indicated that a few vessels exhibited hyperemia (Fig. 2b). No patho-morphological changes in tumour tissue structure were seen in the light-only group.

In the PDT treated animals, necrosis was detected after PDT. Tumours #1–7 showed no or minor amounts (2%) of viable tumour cells remaining (Fig. 2c). The others (#8–9) had 20–60% of viable tumour cells, and #10–11 had 85% and 97% viable cells, respectively demonstrating weak tumour growth inhibition/growth arrest consistent with the volume and TGI results above.

In terms of the microvascular compartment, 9 of 11 mice (#1–9) exhibited severe microvascular damage in the tumours (Fig. 2c), however 2 of these 9 mice (#8 and #9) also exhibited weak damage adjacent to the tumour region (Fig. 2e). The remaining 2 of 11 tumours (#10 and #11) revealed weak microvascular damage throughout (Fig. 2d).

**Figure 3:**
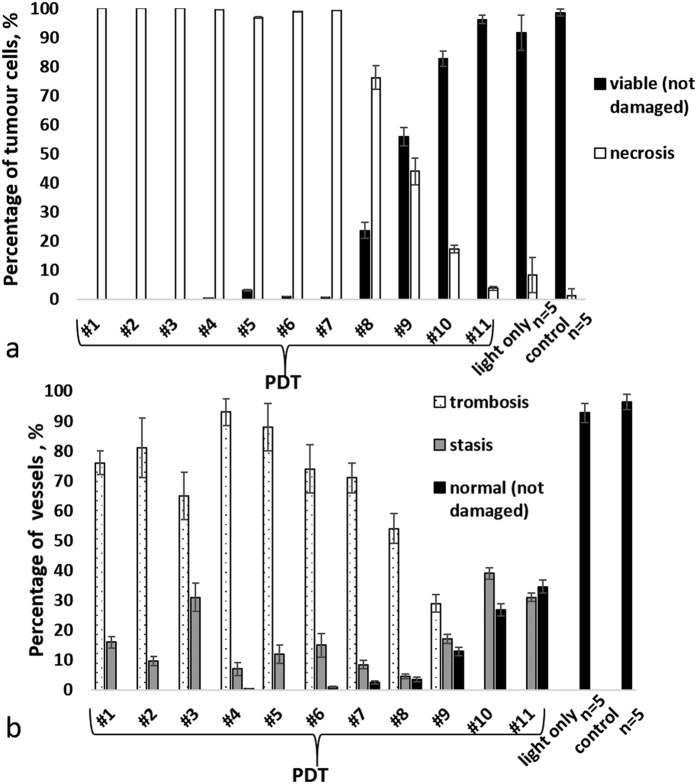
Quantitative assessment of morphological changes of tumour CT-26 at 7 days post PDT: (**a**) percentage of viable and necrotic cells in the tumour; (**b**) percentage of microvascular damages in the tumour. The result are shown as mean ± SD. Notice that in case of complete (necrotic) cell death, thrombosis dominated over the other types of microvascular damages.

Figures 3 demonstrates the quantitative analysis of morphological changes in the tumour cells (Fig. 3a) and microvascular damages (Fig. 3b).

Given this complex and heterogeneous spectrum of volumetric changes and histo-morphological alterations in the tumour PDT response, an alternate sensitive and robust early treatment response indicator *in vivo* would be most desirable.

### Fluorescence intensity and photobleaching of PS studies

**Figure 4:**
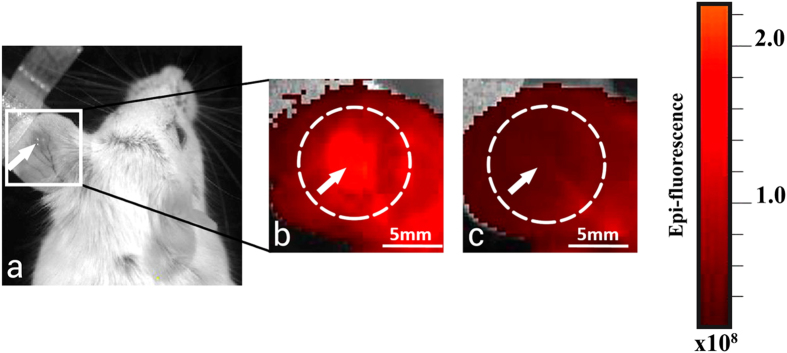
Fluorescence images of ear tumour model CT26. (**a**) White-light photograph of mouse; (**b**) flourescene intensity 1 hour after PS injection (before PDT); (**c**) immediately after PDT (the treatment laser spot size was 5 mm). The tumour is marked by arrow; irradiation field is marked by the white dotted circle. Immediately after PDT, the fluorescence signal in the exposed area declined because of PS photobleaching.

Fluorescence imaging *in vivo* confirmed that Photoditazine accumulated in the CT26 tumour within one hour after intravenous administration (Fig. 4b) and demonstrated PS photobleaching immediately after PDT (Fig. 4c) in all treated tumours.

**Figure 5:**
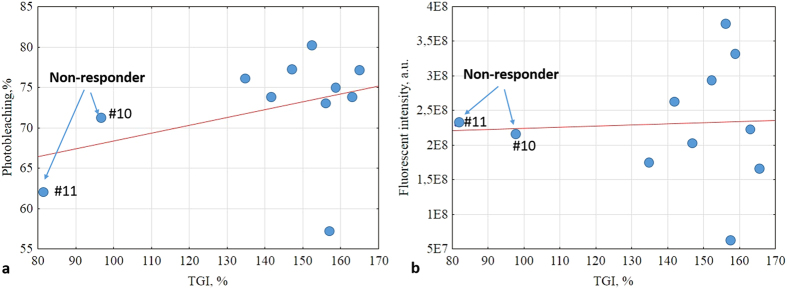
(**a**) Relationship between photosensitizer photobleaching (Equation (1)) and tumour volume quantified by TGI (Equation (2)). A weak correlation is seen (Pearson’s correlation coefficient *r* = 0.386, p = 0.241). (**b**) Fluorescence intensity versus TGI. An even weaker correlation noted (*r* = 0.052, p = 0.879).

Figure 5a shows the relationship between photobleaching (its metric is defined in section PS photobleaching study in Material and Methods) and tumour volume changes, as expressed by the TGI metric. A weak positive trend is seen (Pearson’s correlation *r* = 0.386, p = 0.241). One obvious outlier is seen, but its fluorescence response parameters are indeed correct (as confirmed by repeat measurements); this must be due to inherent biological variability/tumour heterogeneity effects. Figure 5b plots the relationship between overall fluorescence intensity and same tumour volume TGI metric. A very weak correlation was noted (*r* = 0.052, p = 0.879), with same outlier point as in (a). Overall then, neither fluorescence metric seems to be strongly correlated with, and thus predictive of, PDT response as measured by tumour volume changes.

### PDT monitoring with MML-OCA

**Figure 6:**
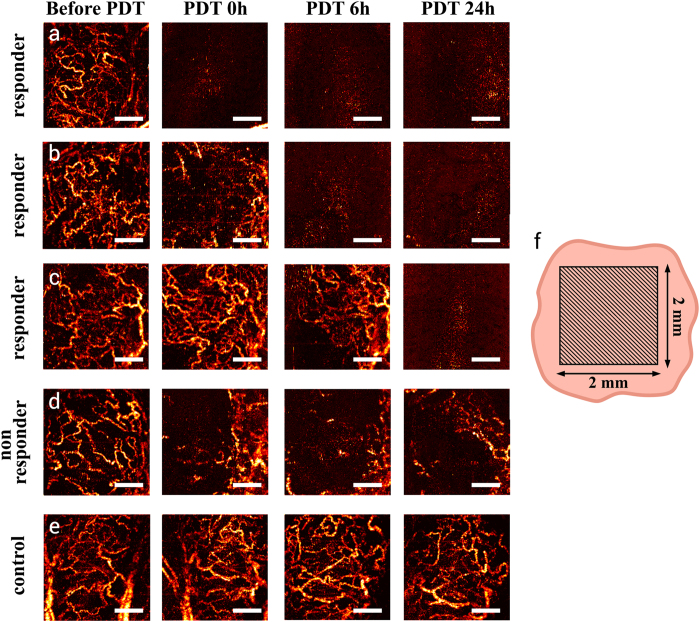
MML-OCA images of microvascular alteration dynamics prior to, immediately following, 6-hrs post, and a day after PDT (100 J/cm^2^, 100 mW/cm^2^). A maximum intensity projection 2D display is shown for ease of comparison, representing a 3D data to a depth of ~1.3 mm. (**a**–**c**) – three separate examples of responding tumours, showing significant microvascular alterations within a day of treatment (or less); (**d**) example of a mildly responding tumour; (**e**) no microvascular changes were noted in the control animal; (**f**) schematic of MML-OCA scanning zone on the tumour (represented in 2D by the flesh-coloured irrelagular contour). Responding tumours’ microvascular inhibition as detected by MML-OCT at t < 24 hrs [(**a**–**c**)] was seen by histology (at t = 7 days) to result from blood vessels thrombosis and hemorrhage (Fig. 2). Scale bar = 500 μm on all images.

Given the variety of tumour growth trajectories, histological findings, and fluorescence responses, it was not surprising that MML-OCA also showed that not all tumours responded to PDT in the same manner. The tumours with severe microvascular damage (#1–9, as revealed by histology at t = 7days), also displayed significant disappearance of blood vessels on MML-OCA within the shorter time interval at t = 1 day. Three of the nine responding tumours (#1–3) exhibited essentially no microvascular network immediately after PDT (Fig. 6a); two tumours (#4 and 5) – at 6 hours after PDT (Fig. 6b); four tumours (#6–9) – at 24 hours after PDT (Fig. 6c). Conversely, histologically-observed weak microvascular damage manifested itself as partial and reversible blood vessels disappearance on MML-OCA. It was observed in two of 11 tumours (#10 and 11) (Fig. 6d). As expected, tumours after light exposure only (Fig. 6e) and untreated tumours demonstrated well-developed blood vessels network on MML-OCA images throughout this observation time period.

Because 9 of 11 treated tumours showed severe microvascular damage within 24 hours of PDT on MML-OCA, this change was chosen as the MML-OCA PDT response metric. Table 1 then summarizes the results of fluorescence imaging, MML-OCA, histology and tumour growth study. If we use TGI as the measure of PDT response, we see that it is associated with percent of viable tumour cells (from histology), does not correlate with photobleaching, and does relate to the disappearance of blood vessels on MML-OCA images within 24hrs of PDT.

It is encouraging that the early-response noninvasive MML-OCA findings correspond well with the later-measured histology trends and with gross tumour volume as quantified by TGI. For the seven tumours that responded well to PDT, OCT-detected microvasculature was essentially obliterated, 7-day histology confirmed severe microvascular damage, and the tumours shrank significantly (TGI was 147–163%). In the two cases of moderate PDT responders (#8 and 9), blood vessels around the tumour showed modest alterations on MML-OCA images, tumour volume effects were less pronounced (TGI was 135 and 142%) percent of viable tumour cells was 20 and 60%, and these tumours may probably regrow. When weak microvascular damages were found by histology at 7 days and blood vessels were still visible on MML-OCA during/after 24 hours post-PDT (#10 and 11), the tumour response was the weakest – the tumour continued to grow (TGI = 97 and 81%) and contained a greater number of viable cells (85 and 97%). Note that the photobleaching data (1^st^ line of Table, Fig. 5) shows no discernable trend and does not seem to distinguish responders from non-responders nor to correlate with final treatment outcome.

## Discussion

In this study, we searched for an early microvascular PDT response criterion via M-mode-like OCT, an approach that allows non-invasive *in-vivo* visualization of tumour blood microvasculature in real time. A thorough analysis of tumour response to PDT with chlorine E6-based photosensitizer Photoditazin by MML-OCA, fluorescence imaging and histology was performed. We found that complete irreversible blood vessels disappearance on MML-OCA images within 24 hours after PDT could be such a criterion. Specifically, MML-OCA can detect thrombosis (one of the main mechanisms of vascular–targeted PDT) due to our employed scanning pattern being sensitive to flowing blood flow only^20^. Accordingly, MML-OCA may provide the opportunity to monitor blood flow interruptions and alterations.

Tumour heterogeneity greatly modulates the efficacy of antitumour therapies, often in unpredictable and poorly understood ways^28^. In our study, the PDT group showed a majority of responders (n = 7), moderate responders (n = 2), but also some non-responders (n = 2). Overall these two non-responders did not seem distinct in their initial sizes, vascular network pattern, or photobleaching characteristics. This diversity of responses to apparently similar treatments is a big problem in PDT and other cancer therapies, which requires more exploration and further underscores the importance of treatment monitoring and patient-specific treatment feedback control and adjustment. Note that previous PDT studies have observed similar puzzling behavior – for example, some non-responding animals were found after PDT with chlorin e6^29^ and with BDP^24^, also with non-obvious reasons for their treatment resistance.

In the present study, PDT was primarily vascularly targeted as the treatment commenced shortly after PS injection when it was still primarily in the blood stream. The histological analysis confirmed the presence of blood vessels damage, as well as signs of cell death (necrosis). Microvascular reactions were divided into severe (irreversible - such as thrombosis, diapedesis hemorrhage, empty blood vessels) and weak (i.e., reversible - sludge, stasis, hyperemia). Thrombosis is the most common microvascular reaction to PDT and appears to be a result of photochemically induced endothelial cell damage^30^. Microvascular thrombosis and stasis lead to hypoxia, often resulting in necrotic tumour cell death. Endothelial cell damage can lead to the establishment of thrombogenic sites within the vessel lumen. This initiates a physiological cascade of responses including platelet aggregation, release of vasoactive molecules, leukocyte adhesion, increases in vascular permeability, and vessel constriction. These effects from damage combine to produce blood flow stasis^31^. In our case, the microvascular damages are believed to be the primary reaction, whereas tumour cells necrosis was the secondary response, likely due to lack of cellular nutrition.

The fluorescence studies yielded largely negative results: Specifically, we found that photosensitizer photobleaching exhibited a weak positive correlation with inhibition of tumour growth, whereas the correlation with fluorescence intensity was even weaker (Fig. 5). Although fluorescence-based methods, aimed at the assessment of the photosensitizer uptake in the tumour (fluorescence intensity) and its PDT ‘action’ (photobleaching quantification), are simple and direct approaches for PDT efficacy evaluation^9^, their applicability for PDT monitoring is still questionable. For example, Cheung *et al*. showed correlation of *in vivo* photosensitizer fluorescence in a tumour and PDT-induced depth of necrosis^32^. Similarly, hotosensitizer uptake measurement prior to light treatment helped to adjust the light dose appropriately and reduce inter-individual variability in response to PDT^33^. Thus some researchers have indeed demonstrated pronounced correlation between PS photobleaching and PDT outcome^34^,^35^. However, it was also shown that photobleaching can predict PDT efficacy only at low concentrations of photosensitizer^35^ and sufficient concentration of oxygen in the tumour tissue^34^. Further, some studies^34^,^36^ reported no association between photobleaching and tumour response to PDT.

Since biological action of PDT depends on oxygen concentration in biotissue, direct measurement of singlet oxygen yield (‘explicit’ metric) was suggested to be an effective method of PDT efficacy estimation^8^,^37^; however such measurements are difficult and time-consuming.

It is known that tumour vasculature is an important target for PDT^38^. Vascular targeting can be further enhanced by chemical modification of the PS molecule that limits its extravasation. Another approach is active targeting of the PS to tumour vascular endothelial markers such as EGFR, SST2R, αvβ3 *integrin* and neuropilin-1^39^,^40^. The use of chlorin-based PS for the vascular-targeted PDT was previously reported^41^,^42^; however, to the best of our knowledge, vascular mechanisms of PDT with Photoditazin have never been explored. We have, however, previously characterized its pharmaco-kinetics and biodistribution in the bloodstream of tumour-bearing mice within 4 hours after *i.v.* injection^43^.

Applying the new MML-OCA approach to derive a microvascular criteria of PDT efficacy, we found that early tumour reaction can be predicted and is essentially independent on the degree of PS photobleachihg during PDT. Namely, even if photobleaching was only partial in some cases, the blood vessels were not visualized 24 hours after PDT in the responding tumours. We attribute this microvascular disappearance to thrombosis and stasis of blood vessels and interruption of blood flow, likely the main factors determining the success of the vascular targeted PDT. The ability of the OCT-based micro-angiography to sensitively detect these microvascular features and quantify their changes makes this approach especially attractive for monitoring PDT treatment efficacy.

Specifically, we show that immediately following and 6–24 hours post PDT, disappearance of the viable (i.e., flowing) microvascular network on MML-OCA (Fig. 6) might predict final tumour necrosis and tumour volume reduction. Yet the situation may be more complex – while one may detect good microvascular response in the tumour regions, “normal” feeding vessels outside the tumour volume may remain undamaged and re-perfuse the successfully treated (and apparently “responding”) tumour regions. This may explain why tumours sometimes shrink initially but then regrow later on, and may explain our results for moderate responders (#8 and 9). In fact, the many studies of PDT microvascular response (e.g.^37^,^44^,^45^, in addition to the ones cited above) concentrate on the tumour regions but tend to ignore the surrounding “healthy” feeder vessels that may often determine the long-term outcome. There may be a case to be made for increasing the PDT irradiation spot size to affect these vessels as well. We will try to explore this effect in our ongoing work, and also delve deeper into the various types of PDT-induced microvascular alterations and blood vessel injuries as detected by MML-OCA.

Approaches that visualize blood regardless of whether it is flowing or stationary (speckle-variance and correlation mapping OCT, photoacoustics, etc) require full elimination of the blood in its liquid state before vessel disappearance is seen on corresponding angiographic images. This will likely occur at higher PDT doses that can cause significant damage of the surrounding normal tissues. In other words, stagnant vessels may already be biologically compromised /dysfunctional at more modest PDT levels, yet still be detected by these angiographic approaches thus giving a false indication of the ineffective treatment. Thus MML-OCA’s ability to detect only flowing/functional microvasculature is a definite advantage for this context. Further, MML-OCA outperforms Doppler OCT in the visualization of flows that are transversal to the scanning beam (since Doppler techniques become severely compromised as flow direction approaches perpendicular orientation) and/or are slow (Doppler effect scales with flow velocity). More technically, Doppler OCT can miss transversal flows because it is particularly sensitive to monotonic change of OCT phase in the vessel regions; yet transversal flows demonstrate chaotic phase variations and often these are filtered out as noise^46^. Here, we again emphasize that MML-OCA visualizes all speckle alterations due to flowing blood that causes the complex speckle variation frequency higher than a pre-selected threshold (chosen to be =96 Hz as the high-pass filter cutoff for this study).

It is worth to mention alternative vasculature-based PDT-monitoring technologies that are not OCT-based. The use of photoacoustics for microvascular PDT-response monitoring has also been examined, suggesting that photoacoustic imaging for PDT may lead to improved efficacy, treatment guidance, and predicting responders *vs* non-responders^17^,^18^. Mallidi *et al*. investigated relationship between PDT efficacy and values of blood oxygen saturation using optoacoustics and showed that 24 hours post PDT these measurements predicted treatment outcome quite well^24^. Diffuse optical spectroscopy was also applied for monitoring PDT; Sunar *et al*. used this method to quantify blood flow, oxygenation and drug photobleaching in PDT-treated cancer patients^47^. Нowever, as revealed in the current study, the key factors are the appearance of thrombosis and stasis of microvessels, so that the ability of the OCT-based micro-angiography to sensitively detect and quantify these effects makes this approach especially attractive for monitoring the treatment efficacy of vascular-based PDT.

## Materials and Methods

### Animals and tumour models

The study was performed on murine colon carcinoma (CT26) transplanted subcutaneously in auricle tissue of mouse strain BALB/c purchased from Nursery for Laboratory Animals, Pushino, Russian Federation (n = 21) at the dose 2 × 10^5^ cells/20 μl of phosphate buffered saline. The specifics of the ear tumour model have been previously described^48^,^49^,^50^. A particular advantage of ear tumour model for OCT investigation is the superficial (intradermal) location of tumour. Its relatively small size allows complete PDT irradiation of the tumour and enables accurate 3D tumour size determination; this is very important for quantifying PDT success. In addition, ear tumours are suitable for angiography research because of their well-developed microvascular network^50^. All experiments were performed in accordance with the European Convention for the Protection of Vertebrate Animals used for Experimental and Other Scientific Purposes (ETS 123) and The Guide for the Care and Use of Laboratory Animals, 8th edition (NRC 2011, National Academic Press). The experimental protocol was approved by the Research Ethics Board of the Nizhny Novgorod State Medical Academy (REB #14, granted December 10, 2013).

### Photodynamic therapy

PDT was performed 10–14 days after tumour inoculation when the linear dimension of tumours reached 3–3.5 mm and blood vessels were visibly well developed. Animals were randomly divided into 3 groups: PDT (n = 11), light only (n = 5) and untreated controls (n = 5). The animals in the PDT group received intravenous injection of a photosensitizer Photoditazin (N-dimethylglucamine salt of Chlorine E6, Veta-Grand, Russia, approved for clinical use) into the tail vein at a dose of 5 mg/kg body weight. One hour after PS injection, when the PS primarily accumulated in the blood vessels as shown by Chen *et al*.^51^, vascular-targeted PDT was carried out. Tumours were exposed to 658 nm light (irradiance rate 100 mW/cm^2^, exposure time 16 min 40 sec, spot size diameter 5 mm, total irradiance 100 J/cm^2^). For PDT procedure, the animals were anesthetized with Zoletil 50 and Rometar 2% (analogue of Xylazin) intramuscularly, as per REB-approved protocol.

### PS photobleaching study

*In vivo* fluorescence imaging was carried out to gauge accumulation of the PS in the tumour and estimate its PDT photobleaching. Epi-fluorescence imaging was performed using IVIS-Spectrum system (Caliper Life Sciences, USA) with excitation at 640 nm (bandwidth 30 nm) and emission at 720 nm (bandwidth 20 nm). The images were acquired before the injection of PS (initial level - autofluorescence), 1 hour after PS administration just prior to treatment (control of Ps accumulation in the tumour), and immediately after PDT (photobleaching). The average fluorescence intensity (FI) in the regions of interest was quantified (Living Image 2.5 software). The background signal before the PS injection was subtracted. Fluorescence intensity was measured in the tumour area. Photobleaching was calculated according to^52^:


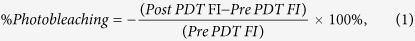


### M-mode-like OCT microvascular visualization

We used a Fourier domain OCT system^53^, with spectral A-scan acquisition rate of 20 kHz, seeded by a superluminiscent diode with central wavelength 1300 nm and 100 nm bandwidth. The setup performs 2D lateral scanning with in-plane range up to 4 mm, allowing acquisition of 3D data sets with 15 μm lateral and 10 μm axial resolution. Scanning system enables a particular predetermined scanning patterns^54^. Microvasculature visualization is based on speckle variation rate of full complex signal with high-pass filtering of OCT B-scans consisting of highly overlapped A-scans, also known as M-mode-like optical coherence angiography (MML-OCA)^20^. Due to the short time lag between A-scans, the displacement of tissue caused by bulk tissue motion artefacts can be significantly compensated. Thus, MML-OCA scanning pattern and corresponding signal processing are more robust to the tissue motions induced by breathing (which can twitch ear tissue) and other possible tissue displacements (up to the speed of several cm/s^20^,^54^).

Microvasculature visualization before and after PDT treatment was performed with a scanning pattern optimized previously^20^,^54^. Briefly, scanning density was 32,768 spectral A-scans per 2 mm in fast scanning direction and 3D data set contained 192 B-scans. Accordingly, the scan time to acquire a single 3D data set was ~5 minutes. To obtain complex A-scans composed of two quadrature components, the reference arm was phase modulated by 90° between A-scan acquisitions. Signal processing^20^,^54^,^55^ filtered out all complex speckle variations with frequencies lower than 96 Hz using uniform high-pass filter. Further, to reduce artifacts arising from the tissue surface effects, we performed amplitude normalization procedure before processing^55^. Because of filtering parameters (96 Hz threshold), this approach visualizes only vessels with flowing blood; stagnant vessels are not detected because stationary blood yields speckle variations with significantly lower frequencies (<50 Hz corresponding to speckle decorrelation time for stationary blood^56^).

### Tumour growth study

Tumours were measured daily in three dimensions with a caliper. The tumour volume V was calculated as: *V* ≈ *π*/6(a·b·c) (mm^3^), where 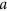 and 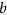 are orthogonal lateral dimensions and *c* is the tumour depth. To characterize tumour growth inhibition (TGI), a coefficient similar to^57^ was used (which included normalization to the initial tumour volume):


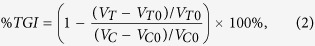


where *V*_*T*_ is the mean tumour volume on day 7 after PDT, *V*_*TO*_ is the mean tumour volume on the day of PDT; *V*_*C*_ and *V*_*CO*_ are the same means for the untreated group. This derived metric quantifies volumetric treatment efficacy while accounting for volume changes due to natural disease progression over the monitoring time interval [TGI > 100% - tumour volume decreased after PDT (effective treatment); TGI ~ 100% - tumour volume did not change, TGI < 100% tumour volume increased (ineffective treatment)]. At 7 days post PDT, animals were sacrificed by cervical dislocation under anesthesia and tissues were harvested for histologic analysis.

### Histology

Verification of the blood vessels reaction and grading of pathological tumour response were carried out by histological examination at t = 7 days after PDT. The excised tumours were embedded in paraffin. Many cross sections were made through the tumour bulk from one side to another. Histological preparations were stained with hematoxylin and eosin (H&E). 7-μm-thick sections were examined with light microscopy on Leica DM1000 system. Several cross-sections from the borders and the center of the tumour were analyzed. Histopathology examination included evaluation of percentage of viable tumour cells (not damaged by PDT) and degree of microvascular damage. Tumour cells without both visible nuclear (karyopyknosis, karyorhexis, karyolysis) and cytoplasm (irreversible dystrophy) injuries were considered to be viable and thus not damaged by PDT. The quantitative assessment of morphological changes in the tumour CT-26 was undertaken via direct cell counting using the grid plate on a Leica DM1000 microscope. Under 40x magnification, the number of grid squares containing the damaged and not damaged (viable) cells and the number of squares containing thrombosis, stasis and normal vessels were counted. The result was presented as a percentage of the total number of grid squares. From each tumour, 3–4 cross-sections were thus examined. >30% of vessels with wall damage (thrombosis, hemorrhage) in the tumour was classified as having severe microvascular damage, <30% of vessel with wall damage was considered to be weak microvascular damage. The 30% categorization value was selected based on preclinical and clinical histo-pathological experience; future work will examine the influence of this particular selection.

### Statistical Analysis

Statistical analysis was performed in Statistica 6.0 software. Pearson’s correlation coefficient was used for comparison between photobleaching of PS and TGI and between fluorescence intensity and TGI. Statistical analysis of tumour growth was performed using the one-tailed Student’s t-test. P values less than 0.05 were considered statistically significant.

## Additional Information

**How to cite this article**: Sirotkina, M. A. *et al*. Photodynamic therapy monitoring with optical coherence angiography. *Sci. Rep.* **7**, 41506; doi: 10.1038/srep41506 (2017).

**Publisher's note:** Springer Nature remains neutral with regard to jurisdictional claims in published maps and institutional affiliations.

## Figures and Tables

**Table 1:** Summary of PDT effects on CT26 tumours.

Animal number	1	2	3	4	5	6	7	8	9	10	11
Photobleaching, %	75	80	77	73	74	77	57	74	76	71	62
Disappearance time on MML-OCA (hrs)	0	0	0	6	6	24	24	24	24	—	—
Microvascular damage (histology)^1^	S	S	S	S	S	S	S	S	S	W	W
Viable tumour cells, % (histology)	0	0	0	0	2	0	0	20	60	85	97
TGI, % (tumour volume metric)	159	152	165	156	163	147	157	142	135	97	81

^a^Classified as severe S (>30% of the vessels exhibited vessel wall damage) or weak W (<30% of the vessels exhibited vessel wall damage). See text for details.
